# A Mini Review on Persulfate Activation by Sustainable Biochar for the Removal of Antibiotics

**DOI:** 10.3390/ma15175832

**Published:** 2022-08-24

**Authors:** Mengxue Li, Peng Li, Qi Zhou, Stephanie Ling Jie Lee

**Affiliations:** 1 College of Environmental Science and Engineering, Tongji University, Shanghai 200092, China; 2 Australian Institute for Bioengineering and Nanotechnology, The University of Queensland, Brisbane, QLD 4072, Australia; 3 College of Chemistry and Chemical Engineering, Anhui University, Hefei 230601, China

**Keywords:** biochar, persulfate, antibiotics, catalytic mechanism, sustainability

## Abstract

Antibiotic contamination in water bodies poses ecological risks to aquatic organisms and humans and is a global environmental issue. Persulfate-based advanced oxidation processes (PS-AOPs) are efficient for the removal of antibiotics. Sustainable biochar materials have emerged as potential candidates as persulfates (Peroxymonosulfate (PMS) and Peroxydisulfate (PDS)) activation catalysts to degrade antibiotics. In this review, the feasibility of pristine biochar and modified biochar (non-metal heteroatom-doped biochar and metal-loaded biochar) for the removal of antibiotics in PS-AOPs is evaluated through a critical analysis of recent research. The removal performances of biochar materials, the underlying mechanisms, and active sites involved in the reactions are studied. Lastly, sustainability considerations for future biochar research, including Sustainable Development Goals, technical feasibility, toxicity assessment, economic and life cycle assessment, are discussed to promote the large-scale application of biochar/PS technology. This is in line with the global trends in ensuring sustainable production.

## 1. Introduction

Antibiotics are used as medicines to protect human and animal health from bacterial infections [1]. The global antibiotics consumption increased from 21.1 to 34.8 billion tons daily from 2000 to 2015 [2]. Based on the chemical structure of antibiotics, the common classes of antibiotics are sulfonamides, tetracyclines, fluoroquinolones, macrolides, and β-lactams [3]. The antibiotics are partially metabolized in vivo, and a significant fraction are released into wastewater with urines and feces. However, the treatment capacity of antibiotics in wastewater treatment plants is limited because of the antibacterial property of antibiotics [4,5]. Residual antibiotics result in the emergence of antibiotic-resistant bacteria, threatening public healthcare [6]. Therefore, it is essential to effectively remove them from wastewater.

Various approaches have been used to remove antibiotics from wastewater, such as biological degradation, adsorption (e.g., plants, materials), membrane filtration, and oxidation processes [7,8,9]. Biological degradation has a low removal efficiency owing to the antibacterial activities of antibiotics. The process of adsorption and membrane filtration can only transfer antibiotics to solid matrices but cannot degrade antibiotics into small molecules [10]. Persulfate-based advanced oxidation processes (PS-AOPs) can induce the generation of highly reactive radicals (sulfate radicals (SO_4_^•−^), hydroxyl radicals (•OH), and superoxide radicals (O_2_^•−^)) to degrade antibiotics into other compounds, water (H_2_O), and carbon dioxide (CO_2_). The persulfates (peroxymonosulfate (PMS) and peroxydisulfate (PDS)) can be activated via ultraviolet light, heat, ultrasound, carbon materials, and transition metals, generating active radicals [11,12]. To overcome the disadvantages of additional energy consumption, high cost, and the problem of metal leaching, the utilization of biochar materials in PS activation has generated increasing attention.

Biochar is usually produced by the pyrolysis of biomass from plants [13,14,15], sludge [16], agricultural waste [17], and wood-processing wastes [18] under anoxic or anaerobic conditions, which can recover and re-utilize the resources in an eco-sustainable manner. Many of the physiochemical properties of biochar, such as high porosity, large surface area, abundant surface functional groups, environmental compatibility, and low cost, favor its usage in PS-AOPs to degrade antibiotics [19,20,21]. To our knowledge, many reviews have focused on the utilization of biochar in soil and water remediation using PS-AOPs technology [11,22,23]. However, they do not specifically consider the removal of antibiotics. It is worth noting that the removal of antibiotics relates to the active sites on the biochar surface, which contribute to PS activation [22]. Moreover, most reviews lack recommendations on future perspectives regarding environmental sustainability to promote the commercialization of biochar.

In this review, a brief overview of the PS activation mechanisms (the radical and non-radical pathways) is provided. The active sites of biochar materials involved in PS activation for antibiotics degradation are summarized. The catalytic activities and activation mechanism of pristine biochar with desirable active sites and biochar with surface modification (non-metal heteroatom-doped biochar and metal-loaded biochar) in PS-AOPs systems are thoroughly discussed. Finally, the existing challenges and future opportunities for the application and commercialization of biochar for antibiotic removal are raised, considering sustainable biochar development regarding Sustainable Development Goals (SDGs), technology, economy and environmental safety aspects. This review provides directions for the design of biochar for the removal of antibiotics in PS-AOPs and supplies constructive recommendations for the development of the biochar/PS system in a scale-up application. The explanation of abbreviations is provided (Table 1) as a quick reference in this review.

## 2. Mechanism of PS Activation

The mechanism of PS activation by biochar materials mainly includes free radical (SO_4_^•−^, •OH and O_2_^•−^) and non-radical pathways (singlet oxygen (^1^O_2_) and surface electron transfer) [24].

The active sites, such as persistent free radicals (PFRs), oxygen-containing groups (C=O, C-OH, C-OOH, C-O-C, etc.), free-flowing electrons in biochar, structural defects, oxygen vacancy, and the redox cycle of metal ions, can activate PS to produce free radicals (Table 2). SO_4_^•−^ with a redox potential of 2.5–3.1 V shows a strong oxidation capacity in comparison with •OH (1.9–2.7 V). SO_4_^•−^ can also react with -OH to form •OH. Biochar materials may deliver an electron to O_2_ to produce O_2_^•−^, which can be an intermediate product for ^1^O_2_ formation [25].

^1^O_2_, as a non-radical reactive species, can be generated in the self-degradation reaction of both PMS and PDS with a slow rate (k = 4.7–5.9 × 10^−2^ M^−1^·s^−1^) [25]. The graphitization of biochar, doped heteroatoms, pyridinic nitrogen (N), graphitic N, oxygen vacancy, and the redox cycle of metal ions and surface groups (C=O, C-OH, etc.) can act as the active sites promoting the formation of ^1^O_2_ (Table 2). The surface electron transfer process, which is related to the active sites of graphitization, N species, defects, and oxygen vacancy (Table 2), is also a non-radical mechanism for biochar in the PS-AOPs. Specifically, biochar catalysts, serving as a transmission medium, facilitate electron transfer from the adsorbed pollutants (electron donor) to metastable PS (electron acceptor) to achieve pollutant oxidation. The non-radical pathway has a lower degradation rate and mineralization extent than the radical pathway due to the relatively weaker oxidation capacity. The non-radical pathway can oxidize organic pollutants at the initial stage, whereas free radicals can further degrade the pollutants into CO_2_ and H_2_O [51,52,53]. Due to its higher selectivity, the non-radical pathway is resistant to various inorganic ions (NO_3_^−^, HCO_3_^−^, halogens, etc.) and background organic matter, which benefits biochar application in natural water.

## 3. Pristine Biochar with Desirable Active Sites for PS Activation to Degrade Antibiotics

Pristine biochar is produced via pyrolysis at >500 °C under inert conditions. Pristine biochar serves as an excellent catalyst for PS activation owning to the high specific surface area, persistent free radicals (PFRs), defects, oxygen-containing functional groups, graphitization properties, and a small quantity of metal components (Table 3).

**Figure 1:**
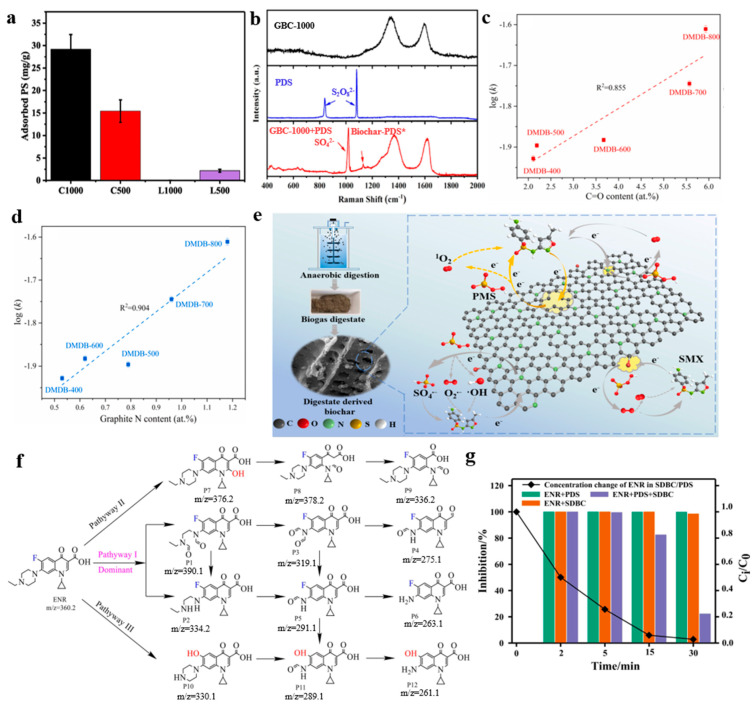
(**a**) The amounts of persulfate (PS) adsorbed on cellulose and lignin–derived biochar prepared at 500 or 1000 °C (C500, C1000, L500 and L1000). Reproduced with permission from [55]. (**b**) In situ Raman spectra of graphitic biochar prepared at 1000 °C (GBC-1000). Reproduced with permission from [49]. Sulfamethoxazole degradation rate in a log (k) versus (**c**) C=O content; (**d**) graphite N content. (**e**) Mechanism scheme of sulfamethoxazole removal in dairy manure digestate–derived biochar (DMDB)/PMS system. Reproduced with permission from [19]. (**f**) Possible degradation pathways of enrofloxacin in sludge–derived biochar (SDBC)/PDS system. (**g**) The toxicity of enrofloxacin and degraded products in an oxidation reaction. Reproduced with permission from [70].

Studies have shown that pollutant degradation may occur on the biochar surface [38,62]. The high specific surface area of biochar can provide sufficient space for the reaction and enhance the loading of active sites and the adsorption capacity for pollutants [63,64,65]. Adsorption of PS on biochar was reported as the essential step for PS activation, due to the facilitated electron transfer between biochar and PS [55]. The adsorption capacity of biochar for PS (Figure 1a) was consistent with the specific surface area, and so was ofloxacin degradation, indicating that the adsorption of PS on biochar may promote PS activation and ofloxacin degradation.

PFRs are free radicals on the surface or interior of biochar, which have a lifetime of hours to months. PFRs can provide electrons to oxidants for organic pollutant degradation, thus endowing pristine biochar with catalytic capacity [66]. As shown by Zhang et al. [26], PFRs were verified on the biochar surface. The PFRs and defects were the active sites for PMS activation to generate radicals (SO_4_^•−^, •OH), which were responsible for the degradation of tetracycline antibiotics. Intrinsic defects could also contribute to the electron transfer non-radical process. Miao et al. [49] used in situ Raman spectroscopy to indicate the formation of metastable surface species (biochar-PDS*) (Figure 1b), which could subsequently oxidize the adsorbed tetracycline via a direct electron transfer pathway [67]. Together with the reduction in the current in chronoamperometry tests after adding PDS or tetracycline solution, electron transfer was achieved in the co-presence of tetracycline molecules and biochar-PDS*. On the surface of biochar, oxygen-containing functional groups with an electron-rich nature are desirable for the mediation and promotion of redox reactions. Wang et al. [19] revealed graphite N and C=O were the active sites using the linear correlations between values of log (k) and active sites (Figure 1c,d). Graphitic N played an essential role in PMS activation by promoting electron transfer from biochar to O_2_ to produce ^1^O_2_ [68]. C=O on the edge of biochar could activate PMS to produce ^1^O_2_ [69]. Together with defects which contributed to the radical pathway and surface electron transfer, free radicals and the non-radical pathways were involved in the biochar/PMS system for sulfamethoxazole degradation (Figure 1e). The existence of metal ions in biochar could facilitate PS activation. Fang et al. [70] reported that the C=O and Fe^2+^/Fe^3+^ on the sludge biochar samples were the main active sites for PDS activation to produce a large amount of ^1^O_2_. ^1^O_2_ was found to be reactive to degrade the tertiary amine of enrofloxacin. Piperazine ring cleavage (P1 and P2), quinolone ring decomposition (P7 and P8), decarboxylation, and defluorination (P10) (Figure 1f) were proposed as the degradation pathways of enrofloxacin. The other products were produced by further oxidization. The toxicity of degraded enrofloxacin products decreased during the oxidation process (Figure 1g).

## 4. Biochar with Surface Modification for PS Activation to Degrade Antibiotics

In order to enhance the catalytic performance of pristine biochar, pristine biochar needs to be treated by functionalization processes. Non-metal heteroatom-doped biochar, and metal-loaded biochar can attach more functional groups to the biochar surface. The abundant active sites, such as PFRs, defects, oxygen-containing functional groups, heteroatom species, metal ions, oxygen vacancy, and graphitized carbon boost the catalytic capacity of biochar for the degradation of antibiotics (Table 3).

### 4.1. Non-Metal Heteroatom-Doped Biochar

**Figure 2:**
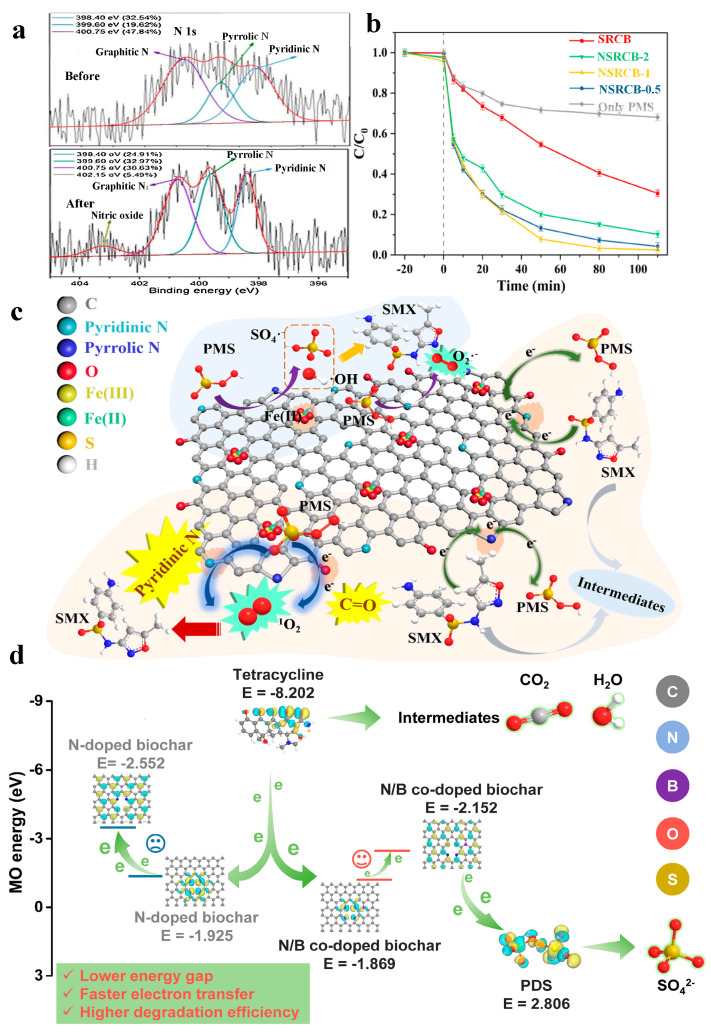
(**a**) High resolution XPS spectrum of N 1s of N–biochar derived from biomass fiber before and after the catalytic reaction. Reproduced with permission from [42]. (**b**) Sulfanilamide degradation by peroxymonosulfate (PMS) activation with biochar materials and (**c**) mechanism scheme. SRCB: sewage sludge–red mud complex biochar. Sulfanilamide: SMX. Reproduced with permission from [40]. (**d**) Electron–transfer pathways driven by different energy gap of biochar materials for tetracycline degradation. Reproduced with permission from [17].

Researchers found that non-metal heteroatom-doped biochar could tune the electronic properties and enhance the catalytic performance of pristine biochar [71]. N atoms can easily substitute the carbon (C) atoms in biochar due to the similar molecular properties to C atoms. N can reconstruct the electron arrangement and enhance the active sites (Pyridinic N, graphitic N, and pyrrolic N) of biochar materials. Ye et al. [42] found that the efficient catalytic performance was due to the facilitated electron transfer originating from graphitization and N functionalization, in which the non-radical pathways (surface electron-transfer and ^1^O_2_) were the dominant pathways for the degradation of tetracycline. Moreover, pyridinic N and graphitic N were the potential active sites for PMS activation (Figure 2a). Liang et al. [40] prepared N-doped biochar from sewage sludge and red mud. Sulfanilamide degradation was enhanced in the N-doped biochar (NSRCBs)/PMS system (Figure 2b), reflecting the important role of N dopants. The as-prepared biochar had abundant catalytic sites for PMS activation to degrade sulfanilamide (Figure 2c). Specifically, the redox cycle of Fe^2+^/Fe^3+^ could generate SO_4_^•−^ and •OH. The pyridinic N could activate PMS to generate O_2_^•−^. The electronic properties of biochar could be regulated by the edge nitrogen. Hence the biochar samples could act as electron mediators to promote electron transfer from sulfanilamide to metastable PMS. Pyridinic N and the C=O group were responsible for the non-radical pathway of ^1^O_2_.

In addition to the N heteroatom, sulfur (S) and boron (B) heteroatoms could also be doped in biochar to improve its catalytic activity. Zhang et al. [39] synthesized N/S-doped moso bamboo biochar. N/S doping increased the concentration of PFRs in biochar, which played the key role in activating PMS. The antibiotic removal efficiency was 92.23%, 88.86%, and 70.97% in 30 min in N-biochar/PMS, S-biochar/PMS, and N/S-biochar/PMS systems, respectively, which were higher than that in the biochar/PMS system (79.86%). The degradation efficiency was consistent with the concentration of PFRs. Dou et al. [17] showed that N and B co-doped biochar not only enhanced the catalytic performance but also switched the radical pathway into the electron transfer-dominated non-radical pathway, as confirmed by theoretical calculations. Due to the narrowest energy gap of N and B co-doping biochar, the electrons of tetracycline (HOMO, −8.202 eV) tended to transfer to BC-PDS* (LUMO, 2.806 eV) via an N/B co-doped biochar bridge (Figure 2d), triggering PDS decomposition without the generation of free radicals. In the catalytic reaction, it was proven that pyridinic N and BC_3_ species were the main contributors in PDS activation to degrade tetracycline.

### 4.2. Metal-Loaded Biochar

**Figure 3:**
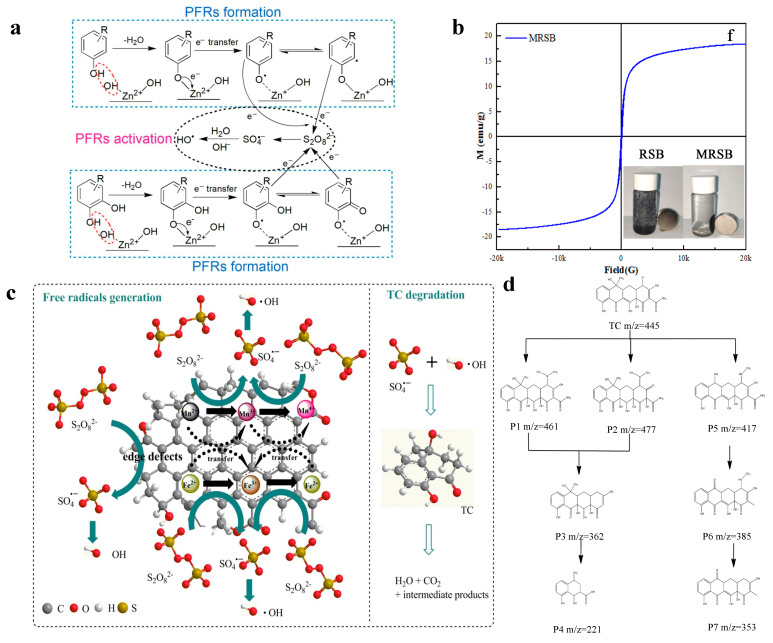
(**a**) Proposed pathway of persistent free radicals (PFRs) formation and persulfate (PS) activation by ZnO/biochar. Reproduced with permission from [13]. (**b**) The magnetization curve of the magnetic rape straw biochar (MRSB). Reproduced with permission from [57]. (**c**) Main catalytic mechanism of PS activation on Mn–doped magnetic biochar for tetracycline degradation. (**d**) Proposed corresponding tetracycline degradation pathways. Reproduced with permission from [59].

Doping with transition metal/metal oxides (Mn, Ce, Cu, ZnO, Cr_2_O_3_, Fe^0^, Fe_3_O_4_, etc.) was also reported to enhance the catalytic performance via synergetic effects [13,72]. The composites can also avoid the aggregation of metal/metal oxides [73] and the potential risk of metal ion leaching. As shown by Xu et al. [13], benefiting from the synergistic effect between biochar and ZnO nanoparticles, more PFRs were created to improve catalytic efficiency. They extrapolated that the molecules with one phenolic hydroxyl, or two adjacent phenolic hydroxyls would produce PFRs through dehydration and electron transport with Zn. Then, PFRs in ZnO/biochar provided electrons to PS to produce SO_4_^•−^ and •OH to remove tetracycline hydrochloride (Figure 3a).

The modified biochar with iron-based compounds (Fe^0^/biochar [27,58], Fe_2_O_3_/biochar [74], Fe_3_O_4_/biochar [37,57], Fe-Mn oxides/biochar [59]) not only enhances the catalytic activity, but also makes the material magnetism to facilitate the recycling of magnetic compounds from water. Huang et al. [57] synthesized a recyclable magnetic rape straw biochar with the saturated magnetization at 18.45 emu/g (Figure 3b). The Fe_3_O_4_ content in the composites promoted PS activation for tetracycline hydrochloride degradation in water via radical and non-radical pathways. Fe^0^ on the surface of biochar acts as an activation center to produce reactive oxygen species for the removal of antibiotics in wastewater. As reported by Ma et al. [58], PDS or dissolved oxygen reacted with Fe^0^ to release Fe^2+^. PDS was activated with Fe^2+^ to form SO_4_^•−^. The part of SO_4_^•−^ transformed to •OH. Fe^2+^ also reacted with dissolved oxygen to generate O_2_^•−^ which could produce ^1^O_2_. Fe^2+^ was oxidized to produce Fe^3+^, which could be reduced by Fe^0^ into Fe^2+^. Moreover, the electron transfer mediated by biochar also facilitated the reduction process of Fe^3+^. Overall, SO_4_^•−^, •OH, O_2_^•−^, and ^1^O_2_ contributed to sulfadiazine degradation. Mn doping in magnetic biochar can further enhance the activation of PS. Huang et al. [59] concluded that the redox cycle of Fe (Fe^2+^/Fe^3+^) and Mn (Mn^2+^/Mn^3+^/Mn^4+^) were present in the catalytic reaction. The results demonstrated that iron–manganese oxide, functional groups, and defects of the composites activated PS to generate free radicals (Figure 3c). Huang et al. also found that tetracycline was degraded through hydroxylation, carbon chain-breaking, ring-opening, demethylation, and hydrogen abstraction (Figure 3d).

Two-dimensional metal materials (layered double hydroxides (LDH), metal–organic frameworks (MOFs)) are used to combine with biochar to improve catalytic capacity synergistically. A biochar scaffold can improve the dispersion and stability of two-dimensional materials and trigger the non-radical route in PS-AOPs [21,75,76,77]. MgFe-LDH/biochar was fabricated using derivatives from the activated sludge and applied for PMS activation to remove aqueous doxycycline [34]. The ^1^O_2_-based non-radical pathway played an important role in doxycycline removal. Biochar-loaded MIL-53(Fe) derivatives (FexC/biochar) with surface area (1685 m^2^/g) were prepared to activate PDS for norfloxacin removal [21]. SO_4_^•−^, •OH, and ^1^O_2_ were involved in the degradation of norfloxacin in the system. Moreover, the FexC/biochar/PDS system exhibited excellent stability in different water bodies.

### 4.3. Non-Metal Heteroatoms and Metal Co-Modified Biochar

Non-metal and metal species can modify biochar to improve its catalytic performance synergistically. Xiao et al. [60] embedded Fe and Ce into N-enriched biochar (Fe/Ce/N-biochar) for PMS activation to remove metronidazole. The second-order rate constant of the Fe/Ce/N-biochar/PMS system was 0.0566 min^−1^, which was 10.5 times greater than that of the biochar/PMS system (0.0054 min^−1^). The Fe and Ce oxides on the biochar served as the centers to activate PMS, while N doping increased the porosity and surface area of biochar, and enhanced the PMS activation capacity. The results revealed that C=O groups and graphitic N in biochar activated PMS via a non-radical pathway, and the redox cycle of Fe (Fe^2+^/Fe^3+^) and Ce (Ce^3+^/Ce^4+^) participated in the radical pathway. Liu et al. [20] focused on the preparation of Fe and N co-doped biochar, due to the improved surface affinity of catalysts by N doping and the high effectiveness, recoverability, and nontoxicity of iron.

## 5. Future Perspectives Considering Sustainability

**Figure 4:**
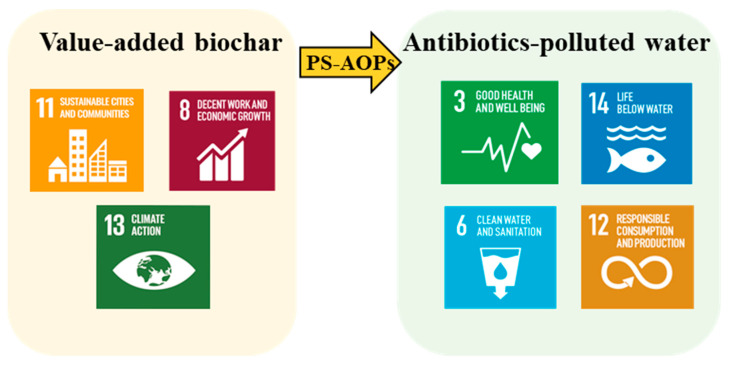
Feasible achievement of the Sustainable Development Goals (SDGs) through the application of the biochar/persulfate (PS) system for the removal of antibiotics.

In 2015, the United Nations General Assembly proposed the Sustainable Development Goals (SDGs), containing 17 items [78]. The sustainable biochar used for antibiotic treatment in PS-AOPs assists in the partial achievement of SDGs (Figure 4). The role of valued-added biochar derived from waste biomass can promote the commercialization of biochar (SDG 11) and the creation of jobs (SDG 8) and provide a strategy for carbon footprint reduction as well as climate change (SDG 13). The biochar/PS technology can degrade antibiotics, thus reducing pollution to life below water and human health (SDG 3, 6 and 14). In addition, the biochar/PS system can serve as an alternative water treatment agent (SDG 12).

A surge of interest has occurred towards the application of biochar to satisfy the sustainability requirements, which benefits the commercialization of biochar products. Although great progress has been made, the following issues need to be addressed in the future to improve the technical feasibility, economic feasibility, and environmental safety.

### 5.1. Technical Aspects

Regarding the large-scale applicability of pristine biochar and modified biochar as discussed above, biochar can be considered as a promising candidate for PS activation to efficiently degrade antibiotics (Table 3). The performance of the biochar/PS system under realistic conditions is identified as an aspect of its sustainability concern, whereas most of the current reports have been conducted in simulated or artificial wastewater rather than actual wastewater. Considering the complexity of actual wastewater, the performance in multi-pollutant systems in actual wastewater needs to be investigated. The efficient catalytic activity is attributed to the abundant active sites in biochar catalysts (Table 2 and Table 3). Novel technologies should be developed to further analyze the role of active sites at the molecular level, to provide direct evidence of the radical and non-radical pathways.

### 5.2. Toxicity Assessment

Toxicity assessments during the production and application of biochar are critical to satisfy sustainability. Risks to occupational health and safety can occur through the exposure via dermal, oral, inhalational, and ocular routes [79]. Biochar may contain toxic compounds, such as heavy metals or organics (polycyclic aromatic hydrocarbons and polychlorinated aromatic compounds, etc.) [80]. The contents of the toxic matters generally depend on the feedstock and preparation methods [14,56,81]. However, to date, the toxicity analysis of biochar catalysts used in biochar/PS system has not been well studied and documented.

Highly toxic intermediates can be generated in the water treatment process [82,83]. For example, the intermediate (*m*/*z* 477.15) of tetracycline was deemed as the molecule leading to the toxicity of the solution [84]. Additionally, 4-nitro-sulfamethoxazole as one of the sulfamethoxazole transformation products was found to be carcinogenic and mutagenic [85]. Few researchers analyzed the toxicity of products from antibiotics degradation in the biochar/PS system. For instance, Wang et al. [86] found that sulfamethoxazole significantly affected the growth of Chinese cabbage seed root. However, the N-biochar/PMS system efficiently reduced the phytotoxicity of sulfamethoxazole and its degradation products. Xiao et al. [60] evaluated the exposure risk of the degradation products of metronidazole in the Fe/Ce/N-BC/PMS system using 3-(4,5-dimethyl-2-thia-zolyl)-2, 5-diphenyl-2H-tetrazolium bromide (MTT) assay (A375 skin cell line). The results showed that most cells survived in the reaction solutions. Accordingly, the ecotoxicity of biochar and degradation intermediates are indispensable research topics for the application of the biochar/PS system in the future.

### 5.3. Economic and Life Cycle Assessment

Consideration of the costs in the production of biochar and application of the biochar/PS system is important for commercialization. The main production costs refer to biomass collection, chemicals, electricity, equipment maintenance, transportation, labor, and so on. Du et al. [87] calculated the economic value of biochar prepared via pyrolysis at 550 °C. The biochar yields were 42.34%. The net production cost of the biochar was estimated to be USD 0.18/kg. However, surface modifications of biochar with other chemicals are required to promote PS activation (Table 3), leading to increased production costs of the derived catalysts. Cheng et al. [88] assessed the production costs of biochar activated with potassium hydroxide (KOH). Considering the costs of biomass, KOH, electricity, and transport, the total cost of the biochar was USD 9.82/kg, which was lower than that of activated carbon (~USD 45.71/kg) sold in Henan Huasheng Charcoal Industry Ltd. (Henan, China), but higher than that of unmodified biochar. Future studies are expected to find ways to reduce the production costs of the functionalized biochar to satisfy the sustainability considerations [89]. It is also necessary to predict the cost of the biochar/PS system in application. Wang et al. [90] evaluated the cost of sodium persulfate (USD 3.87/kg) and sludge-derived biochar (USD 0.222–0.584/kg). Their calculation of biochar/PS system for treating 1 L membrane bio-reactor effluent with biochar of 5 g/L and PS of 150 mM was less than USD 0.16. Within this context, biochar can be considered as a cheap and cost-effective option for treating antibiotic-polluted water in PS-AOPs, though more studies are required to further enhance the economic feasibility of biochar materials.

Concerns have been raised in relation to the environmental sustainability of a newly proposed technology, rather than focusing solely on technical efficiency. Life cycle assessment (LCA) is one of the major tools for the analysis of environmental impacts throughout the whole life cycle of a product/process [91]. Huang et al. [92] compared the Fenton oxidation of trichloroethylene by biochar synthesized at various microwave power levels (200–400 W). According to the removal efficiency and LCA results, the microwave power level of 300 W induced the highest catalytic effect and the lowest environmental impact. However, the LCA evaluation for the use of biochar in PS-AOPs is still missing in published reports. 

## 6. Conclusions

This review summarized biochar for the treatment of antibiotic-polluted water in PS-AOPs. The radical (SO_4_^•−^, •OH and O_2_^•−^) and non-radical (^1^O_2_ and surface electron transfer) processes contribute to the degradation of antibiotics. The high specific surface area of biochar and the active sites on the biochar surface (PFRs, oxygen-containing groups, defects, sp^2^ carbon network, heteroatoms, the redox cycle of metal ions, and oxygen vacancy) are responsible for PS activation to remove antibiotics. Generally, the biochar applied in the PS-AOPs is divided into pristine biochar, non-metal heteroatom-doped biochar, metal-loaded biochar, and non-metal heteroatoms and metal co-modified biochar. Pristine biochar has a relatively low catalytic efficiency, which is related to the limited active sites. The contents of active sites can be regulated through heteroatom doping and metal loading, thus enhancing the catalytic performances of pristine biochar. The synthesis and application of biochar materials can be a sustainable option for converting biomass waste into value-added biochar, resulting in the partial achievement of SDGs. The sustainable prospective of biochar applied in PS-AOPs for the treatment of antibiotics is also discussed, considering the technical aspects, toxicity assessment, and economic and life cycle assessment, to promote the further development of biochar materials. Thus, this review will improve the basic understanding of utilization of biochar in PS-AOPs to remove antibiotics and provide preliminary guidance for real-word application and commercialization of biochar products.

## Figures and Tables

**Table 1:** List of abbreviations.

Abbreviations	Full Names
PS-AOPs	Persulfate-based advanced oxidation processes
PS	Persulfate
PMS	Peroxymonosulfate
PDS	Peroxydisulfate
SO_4_^•−^	Sulfate radicals
•OH	Hydroxyl radicals
O_2_^•−^	Superoxide radicals
^1^O_2_	Singlet oxygen
H_2_O	Water
CO_2_	Carbon dioxide
SDGs	Sustainable Development Goals
PFRs	Persistent free radicals
N	Nitrogen
C	Carbon
S	Sulfur
B	Boron
LDH	Layered double hydroxides
MOFs	Metal–organic frameworks
MTT	3-(4,5-dimethyl-2-thia-zolyl)-2, 5-diphenyl-2H-tetrazolium bromide
KOH	Potassium hydroxide
LCA	Life cycle assessment

**Table 2:** Active sites on biochar catalysts for PS activation to remove antibiotics.

Mechanism		Active Sites
A free radical pathway	SO_4_^•−^, •OH	PFRs [26], oxygen-containing groups (C=O, C-OH, C-OOH, C-O-C, etc.) [27,28,29,30], sp^2^ hybridized carbon network [31], the redox cycle of metal ions [32,33], defects [34,35]
	O_2_^•−^	PFRs [30], the redox cycle of metal ions [36], oxygen vacancy [20], defects [34], C=O [37]
A non-radical pathway	^1^O_2_	Graphitization [38], heteroatom doping [39], pyridinic N [40,41], graphitic N [42], oxygen vacancy [43,44], the redox cycle of metal ions [32,33], C=O [16], C-OH [28,45]
	Surface electron transfer	Graphitization [46,47], graphite N [42], pyridine N, pyrrolic N [48], defects [49], oxygen vacancy [50]

**Table 3:** Application of biochar catalysts in PS-AOPs for the removal of antibiotics.

Catalysts	Biomass	Antibiotics	Attached Functional Groups	Pyrolysis Temperature (°C)	Specific Surface Area (m^2^/g)	Active Sites	Mechanism	Refs.
Pristine biochar	Poplar and pine sawdust	Tetracycline, Chlortetracycline, Doxycycline	/	500–900	76.6–405	PFRs, defects	SO_4_^•−^, •OH	[26]
	Coconut shell, eucalyptus leaves, walnut shell	Quinolone		500	/	PFRs	SO_4_^•−^, •OH	[54]
	Sludge	Tetracycline		900	537	Graphite N, C=O	O_2_^•−^, ^1^O_2_, electron transfer	[16]
	Digestate	Sulfanilamide		800	252	Graphite N, defects, C=O	SO_4_^•−^, •OH, ^1^O_2_, electron transfer	[19]
	Wood chip	Sulfamethoxazole		900	298	Graphitic carbon, C=O	SO_4_^•−^, •OH, O_2_^•−^, ^1^O_2_, electron transfer	[18]
	Cellulose, lignin	Ofloxacin		500–1000	5.74–58.9	Defects	^1^O_2_	[55]
	Wood chips	Tetracycline		1000	600	Defects	Electron transfer	[49]
	Sludge	Ciprofloxacin		700	52.7	Fe^0^/Fe^2+^/Fe^3+^, N, O	SO_4_^•−^, •OH, ^1^O_2_	[56]
	Hyperaccumulators	Ciprofloxacin		700	973	Graphitic N	Electron transfer	[14]
Modified biochar	Pinewood	Ciprofloxacin	N	800	1398	Pyridinic N, C=O	SO_4_^•−^, •OH, ^1^O_2_, electron transfer	[41]
	Moso bamboo	Oxytetracycline, Chlortetracycline, Pefloxacin	N, S	300–600	104	PFRs, heteroatom doping	SO_4_^•−^, •OH, O_2_^•−^, ^1^O_2_	[39]
	Wheat straw	Tetracycline	N, B	700	45	Pyridinic N, BC_3_	SO_4_^•−^, •OH, electron transfer	[17]
	Plant	Tetracycline hydrochloride	ZnO	400	140	PFRs	SO_4_^•−^, •OH	[13]
	Chrome shavings	Tetracycline	Cr_2_O_3_	400/800	1578	PFRs, defects, Cr^3+^/Cr^4+^, C-O-C, C=O, pyridinic N, oxidized N	SO_4_^•−^, •OH, O_2_^•−^, ^1^O_2_	[35]
	Rape straw	Tetracycline hydrochloride	Fe_3_O_4_	400	14.6	Fe^2+^/Fe^3+^, C=O	SO_4_^•−^, •OH, O_2_^•−^, ^1^O_2_	[57]
	Red mud	Sulfadiazine	Fe^0^	800	119	Fe^0^/Fe^2+^/Fe^3+^, C=O	SO_4_^•−^, •OH, O_2_^•−^, ^1^O_2_	[58]
	Eggshells	Norfloxacin	Co-Ca bimetallic oxide	400/900	1.22	Co^3+^/Co^2+^, oxygen vacancy	SO_4_^•−^, •OH, ^1^O_2_	[44]
	Bamboo	Tetracycline	Fe-Mn bimetallic oxide	900	365	Mn^2+^/Mn^3+^/Mn^4+^, Fe^2+^/Fe^3+^, oxygen functional groups, defects	SO_4_^•−^, •OH, electron transfer	[59]
	Banyan branch	Metronidazole	Fe, Ce, N	800	225	Fe^2+^/Fe^3+^, Ce^3+^/Ce^4+^, graphitic N, C=O	SO_4_^•−^, •OH, O_2_^•−^, ^1^O_2_	[60]
	Glucose	Tetracycline	Cu, N	700	352	Cu^2+^/Cu^3+^	•OH, electron transfer	[61]
	Sludge	Doxycycline	MgFe-LDH	500/150	84.7	Fe^2+^/Fe^3+^, defects, C=O	SO_4_^•−^, •OH, O_2_^•−^, ^1^O_2_	[34]
	Bamboo chips	Norfloxacin	MIL-53(Fe)	450/900	1685	Fe^0^/Fe^2+^/Fe^3+^, defects, graphitized carbon	SO_4_^•−^, •OH, ^1^O_2_	[21]

## Data Availability

Not applicable.
